# Parity–time-symmetric photonic topological insulator

**DOI:** 10.1038/s41563-023-01773-0

**Published:** 2024-01-09

**Authors:** Alexander Fritzsche, Tobias Biesenthal, Lukas J. Maczewsky, Karo Becker, Max Ehrhardt, Matthias Heinrich, Ronny Thomale, Yogesh N. Joglekar, Alexander Szameit

**Affiliations:** 1 Institute of Physics, University of Rostock, Rostock, Germany; 2 Institut für Theoretische Physik und Astrophysik, Julius-Maximilians-Universität Würzburg, Am Hubland, Würzburg, Germany; 3 Department of Physics, Indiana University–Purdue University Indianapolis (IUPUI), Indianapolis, IN USA; 4 Department of Life, Light and Matter, University of Rostock, Rostock, Germany

**Keywords:** Micro-optics, Topological insulators

## Abstract

Topological insulators are a concept that originally stems from condensed matter physics. As a corollary to their hallmark protected edge transport, the conventional understanding of such systems holds that they are intrinsically closed, that is, that they are assumed to be entirely isolated from the surrounding world. Here, by demonstrating a parity–time-symmetric topological insulator, we show that topological transport exists beyond these constraints. Implemented on a photonic platform, our non-Hermitian topological system harnesses the complex interplay between a discrete coupling protocol and judiciously placed losses and, as such, inherently constitutes an open system. Nevertheless, even though energy conservation is violated, our system exhibits an entirely real eigenvalue spectrum as well as chiral edge transport. Along these lines, this work enables the study of the dynamical properties of topological matter in open systems without the instability arising from complex spectra. Thus, it may inspire the development of compact active devices that harness topological features on-demand.

## Abstract (web summary)

The authors propose a non-Hermitian topological insulator with a real-valued energy spectrum based on a periodically driven Floquet model implemented in a photonic platform where generalized parity–time symmetry is protected against spontaneous symmetry breaking under a spatiotemporal gain and loss distribution.

## Main

The discovery of the quantum Hall effect^1^ shed the first light on the role of topology in correlated electron systems, and thereby inspired the subsequent emergence of topological insulators^2–6^ as one of the most active current areas of research across a variety of fields in physics. As an independent class of materials in their own right, these systems are characterized by topologically protected transport along their boundary that is robust against defects and disorder. By contrast, their bulk typically remains entirely insulating or features substantially reduced rates of wave packet diffraction. In recent years, topological insulators have been experimentally realized and studied on a wide range of different physical platforms^7–14^. All of these implementations, however, have Hermiticity in common, as they inherently assume closed systems, and their dynamics can therefore be described entirely independently of global attenuation or amplification. Yet, non-Hermiticities are omnipresent in realistic physical systems due to interactions with the environment. Although the energy exchange associated with such coupling to a reservoir in general gives rise to exponentially decaying or amplified states, certain symmetries may keep such instability at bay: as was shown by Bender and Böttcher in 1998, parity–time-symmetric (PT-symmetric)^15^ configurations can globally exhibit real eigenvalue spectra despite featuring a non-zero imaginary part of the potential landscape. As it turns out, these properties are not restricted to a combination of parity flip and time reversal, but can in fact be generalized to all self-inverse antiunitary operators^16^. The notion of PT symmetry encountered particularly fertile ground in photonics, where the imaginary part of potentials can be readily implemented as gain and loss for electromagnetic waves. In conjunction with the refractive index that represents the potential’s real part, light-based settings enabled the experimental exploration of PT-symmetric systems and their peculiar features, ranging from non-orthogonal eigenmodes to the emergence of exceptional points^17–24^ at the phase transition that marks the spontaneous breaking of this complex symmetry. More recently, efforts to combine the two previously separate realms of topology and non-Hermiticity have resulted in an extensive topological classification of non-Hermitian symmetries^25^ as well as important advances ranging from topological insulator lasers^26^ and their acoustic counterparts^27^ to light steering along interfaces between dynamically defined amplifying and attenuating domains^28^, topological funnelling of light^29^ and the direct measurement of a non-Hermitian topological invariant^30^. Notably, although PT symmetry and topological insulators were at first considered to be mutually exclusive^31^, subsequent experiments in one-dimensional photonic structures^32,33^, electronic circuits^34^ and mechanical metamaterials^35^ nevertheless showed that non-trivial topological features can indeed be found in certain PT-symmetric arrangements, and even tuned by means of nonlinearity^36^. However, a key obstacle was found to be the selective breaking of the PT symmetry in chiral boundary states^37^. There are theoretical proposals at hand to overcome the issue^38,39^. However, they are based on requirements that are challenging to implement on integrated-optical platforms, such as non-Hermitian hopping terms or precisely tuned continuous spatiotemporal gain–loss distributions. As a result, the experimental realization of a genuine PT-symmetric topological insulator remains elusive to this day.

**Fig. 1:**
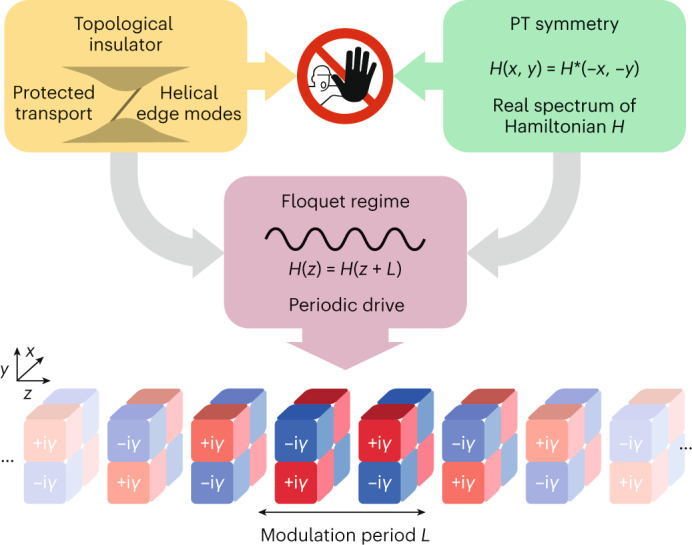
Conceptual idea of a PT-symmetric topological insulator. Conventional wisdom regards topological insulators and PT symmetry as mutually exclusive concepts. Distributing gain and loss (±i*γ*, indicated as red (+) and blue (–), respectively) dynamically along the spatial degrees of freedom *x* and *y* and the evolution coordinate *z* of a periodically modulated system allows for the construction of a complex Floquet drive that overcomes this dichotomy by simultaneously supporting topologically protected edge transport and an entirely real eigenvalue spectrum in a genuinely non-Hermitian arrangement.

In this work, we theoretically propose and experimentally demonstrate a non-Hermitian topological insulator with an entirely real spectrum. In contrast to conventional static implementations of PT symmetry that arrange gain and loss spatially^23–35^, we construct a periodically driven Floquet model that distributes the non-Hermitian components dynamically in both space and time (compare with Fig. 1). In particular, we employ a generalized PT-symmetric extension of a ${{\mathbb{Z}}}_{2}$ topological insulator^40^ and implement the constituent steps of its anomalous Floquet driving protocol^41–44^ in a mesh-like arrangement of selectively coupled optical wave guides. This approach allows us to overcome the limitations discussed in the literature^31^ and realize a system with a novel type of topological boundary states. Notably, in this dynamical non-Hermitian arrangement, the system’s bulk as well as edge states are protected from instability that would typically be induced by coupling to the environment and preclude a lasting transport along the edge, as evidenced by the fact that the quasi-energy band structure remains entirely independent of it (Supplementary Section 2).

**Fig. 2:**
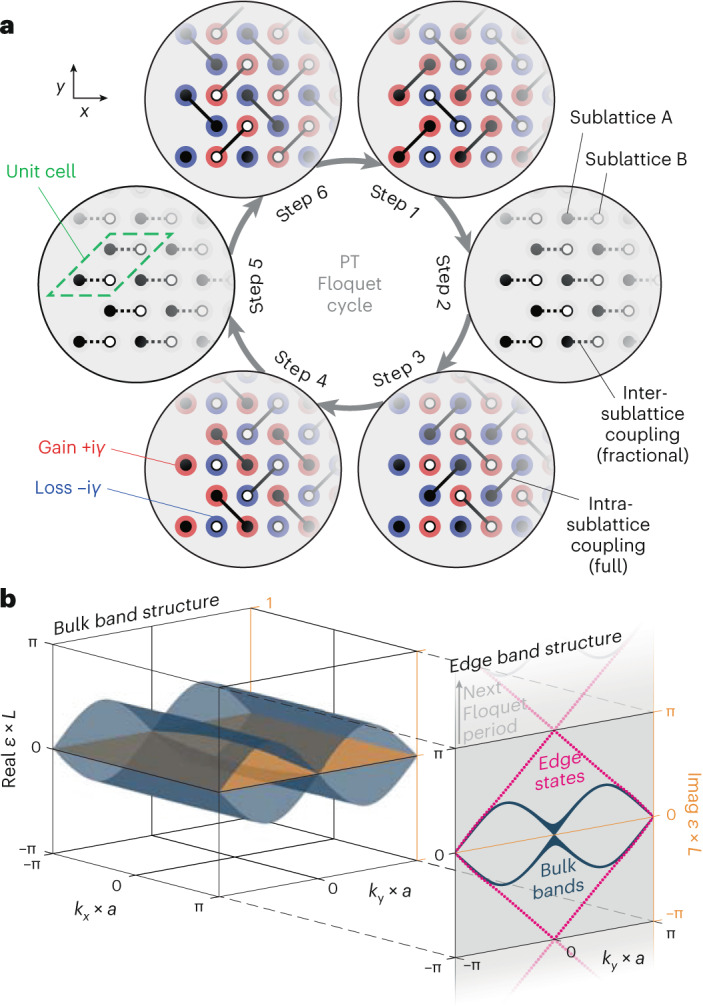
Non-Hermitian Floquet driving protocol and band structure. **a**, As a PT-symmetric generalization of the anomalous ${{\mathbb{Z}}}_{2}$ drive^40^, our protocol consists of six distinct steps in which individual pairs of sites are allowed to interact. Note that only excitations in the two sites residing near the obtuse-angle (135°) corners of the rhombic unit cell (marked by a green dashed outline) populate the chiral edge states of the driven lattice, whereas excitations of the other two sites near the acute-angle (45°) corners result in closed loops. The individual sites of the two sublattices, A and B, are indicated by black and white-filled circles, respectively, while the presence of gain and loss is highlighted by red and blue haloes, respectively. **b**, The left side shows the numerically calculated bulk band structure as function of the quasimomenta *k*_*x*_ and *k*_*y*_ for full intra-sublattice coupling (solid connecting lines in steps 1, 3, 4 and 6 of **a**) and 66% inter-sublattice coupling (dashed connecting lines in steps 2 and 5 of **a**). Here, *a* denotes the lattice constant. The blue-shaded surface represents the real part of the quasi-energy *ε*. The intact PT symmetry of the arrangement is evidenced by the globally vanishing imaginary (Imag) part (orange). The right side shows that in addition to the projection of the bulk bands (blue), the edge band structure (numerically calculated for a semi-infinite ribbon) exhibits a pair of dispersion-free counter-propagating chiral edge states (dotted magenta lines) that likewise feature entirely real eigenvalues.

In the tight-binding limit, light evolves in our two-dimensional photonic structure in accordance with the discretized paraxial Helmholtz equation (Supplementary Section 1):

1 $$\mathrm{i}\frac{{\mathrm{d}}}{{{\mathrm{d}}z}}{{{E}}}_{m}\left(z\right)={g}_{m}\left(z\right){{{E}}}_{m}\left(z\right)+\sum _{l\in \left\langle m\right\rangle }{c}_{l,m}\left(z\right){{{E}}}_{l}\left(z\right)$$

for the electric field amplitude *E*_*j*_ at lattice site *j*. It is formally equivalent to the Schrödinger equation with the third spatial dimension *z* playing the role of time^45^, which is why, in the following, we will refer to *z* as time. The transverse dynamics are represented by the sum over the nearest neighbours 〈*m*〉 of site *m*. The driving protocol itself is periodic along *z* with a Floquet period of *L*, and the on-site potentials *g*_*m*_(*z*) are piecewise constant for six discrete steps of equal length *L*/6. Similarly periodic Hermitian couplings *c*_*l*,*m*_(*z*) are chosen such that, in each step, only specific pairs of nearest neighbours interact (Fig. 2). The full dynamics of the system are therefore described by the Floquet operator after one period:

2 $$U\left(L\right)={U}_{6}{U}_{5}{U}_{4}{U}_{3}{U}_{2}{U}_{1}={{\mathrm{e}}}^{-\mathrm{i}{H}_{\text{eff}}L}$$

with the effective Hamiltonian *H*_eff_ and ${U}_{n}={{\mathrm{e}}}^{-\mathrm{i}{H}_{n}L/6}$ with the (static) single-step Hamiltonians *H*_*n*_. The explicit forms of these operators are provided in Supplementary Tables 1 and 2. The eigenvalues e^–i*εL*^ of the Floquet operator yield the quasi-energies *ε* that, due to the cyclic driving protocol, are periodic^43^ with 2π/*L*. For real-valued on-site potentials *g*_*m*_(*z*), the Floquet operator is by definition unitary, and the effective Hamiltonian (compare with equation (2)) is Hermitian ${H}_{\text{eff}}^{* }={H}_{\text{eff}}^{{\mathrm{T}}}$, where * denotes complex conjugation and T denotes transposition. Similar to the model discussed in the literature^46,47^, the unit cell of our lattice comprises a total of four sites belonging to two distinct sublattices (indicated in Fig. 2a as black for sublattice A and white for sublattice B). Note that while inter-sublattice couplings are crucial for the desired photonic ${{\mathbb{Z}}}_{2}$ topological insulator^40^ to be established, they are confined to steps 2 and 5, while all remaining steps exclusively promote interactions within each of the two sublattices. In this vein, the protocol gives rise to a pair of counter-propagating boundary states that are topologically protected by virtue of fermionic time reversal symmetry defined by

3 $${\mathcal{T}}{{H}^{* }\left(z\right){\mathcal{T}}}^{-1}=H\left(L-z\right),$$

where ${\mathcal{T} (\mathcal{T}^{-1})}$ denotes the (inverse) time reversal operator and fulfils ${\mathcal{T}}{{\mathcal{T}}}^{* }{\mathbb{=}}{\mathbb{-}}{\mathbb{1}}$.

To extend this Hermitian Floquet system in a PT-symmetric fashion, we introduce complex on-site potentials $g\left(z\right)\to \pm \mathrm{i}\gamma \left(z\right)$ whose imaginary part ±*γ* encodes gain (+) as well as loss (−) with equal magnitudes. As a result, the system’s Hamiltonian is no longer Hermitian, that is H* ≠ *H*^T^, and as such allows for two distinct variants of time reversal symmetry: the conventional one using complex conjugation (equation (3)), and a different one relying instead on transposition:

4 $${\mathcal{T}}{{H}^{{\mathrm{T}}}\left(z\right){\mathcal{T}}}^{-1}=H\left(L-z\right).$$

While both of these non-Hermitian symmetries can in principle support a pair of topologically protected boundary states^25^, the transposition-based approach detailed in equation (4) readily allows for the non-Hermitian contributions to be placed in coupling steps 1, 3, 4 and 6 (Fig. 2a) where only intra-sublattice couplings occur. Such an alternating and step-wise balanced arrangement ensures that whenever one of the sublattices experiences gain, the other one is subject to loss (Fig. 2a). Importantly, despite the genuinely non-Hermitian single-step Hamiltonians and the accordingly non-unitary Floquet operator, the quasi-energy spectrum of both the bulk and the boundary states remains entirely real for arbitrary values of *γ* (Fig. 2b). This is a direct result of the connection of the non-unitary Floquet operator *U*(*γ* ≠ 0, *L*) to its unitary counterpart *U*(*γ* = 0, *L*), which can be written as *U*(*γ* = 0, *L*) = *M*(*γ*)*U*(*γ*, *L*)*M*^–1^(*γ*), with *M*(*γ*) = Diag(exp(2*γ*), 1, exp(2*γ*), 1), in line with the time reversal symmetry and the temporal distribution of the gain and loss. Consequently, the non-Hermitian system strictly retains both the real eigenvalue spectrum and the topological properties of the Hermitian case, despite undergoing an unequivocally non-unitary time evolution that distinguishes it from its Hermitian counterpart^40^. In other words, the proposed arrangement has only an unbroken PT-symmetric phase regardless of the applied contrast between gain and loss. A detailed analysis of the properties of the Floquet operator and the generalized PT symmetry of the effective Hamiltonian is provided in Supplementary Section 2. Due to the non-orthogonality of the eigenstates of the Floquet operator, the intensity of any given state is no longer a conserved quantity but rather oscillates along *z* (Supplementary Section 4 and Supplementary Fig. 3)—a well-known and crucial dynamical feature of PT-symmetric systems^17^.

**Fig. 3:**
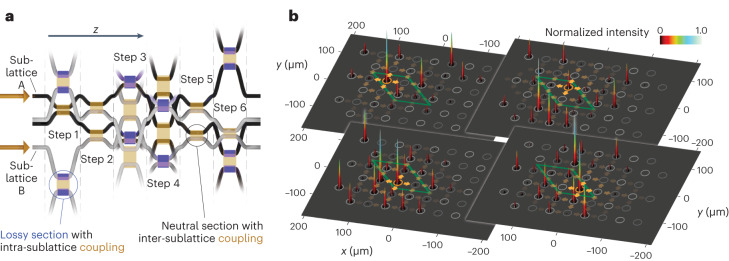
Photonic implementation and bulk excitations. **a**, Schematic of the three-dimensional wave guide mesh lattice that implements the proposed protocol in an entirely passive setting. Shown is one transverse unit cell as well as the sections of wave guides from the adjacent unit cells it interacts with in the course of the driving cycle. The sublattices A and B are indicated by the respective colours (dark/light grey) of the wave guides, and the regions with deliberately introduced losses are shaded purple. More details are provided in Supplementary Fig. 2. **b**, Experimentally observed intensity output patterns at the output facet of the sample resulting from single-site bulk excitations in a lattice composed of 3 × 4 unit cells. As a guide to the eye, positions of the wave guides of this lattice are indicated by grey circles, whereas the four different initially excited wave guides of a bulk unit cell (green rhombus) are highlighted with orange circles. Note that in each case, the wave packets are subject to substantial bulk diffraction (highlighted by the orange arrows) enabled by the fractional inter-sublattice couplings in steps 2 and 5 of the driving protocol. Simulations of the degree of bulk localization for different inter-sublattice couplings are provided in Supplementary Fig. 4. Source data

To experimentally probe the topological transport characteristics of our system, we employed the femtosecond laser direct writing technique^48^ to fabricate wave guide lattices composed of 4 × 3 unit cells in the transverse (*x*, *y*) plane and two full Floquet cycles along *z* in a 150-mm-long fused silica sample. In each step of the driving protocol, the respectively interacting wave guides are brought into close proximity to one another via sinusoidal bends so as to facilitate the desired fraction of light to be transferred between them while suppressing interactions between all other lattice sites^43^. Along these lines, the four intra-sublattice couplings (steps 1, 3, 4 and 6) are set to fully transfer light from one wave guide to the next to ensure a maximum degree of helicity. By contrast, the couplers for steps 2 and 5 have to be designed for a fractional transfer between the sublattices, as perfect coupling (or full transfer of light) in these components would render the Floquet operator trivially unitary (Supplementary Section 3). Along these lines, an inter-sublattice transfer ratio of (67.8 ± 0.75)% was implemented in the experiments. In turn, the non-Hermitian features of the system were established by introducing losses via a multiplicity of microscopic scattering centres deliberately created during the inscription process^23^. These microscopic points scatter some light away from the wave guide such that it is effectively lost to the environment. In this fashion, we systematically shifted the imaginary part of the spectrum to allow for an entirely gain-free or passive implementation that faithfully reproduces the PT-symmetric characteristics of the gain/loss arrangement while avoiding the thermal noise associated with net-gain regions^49^. Note that steps 2 and 5 are realized without additional losses, and, in the co-moving dampened frame, this corresponds to an effective gain. This absence of true gain faithfully preserves the characteristic dynamics of the system while allowing for an entirely passive experimental implementation. The distribution of the lossy sections within one Floquet unit cell of the wave guide mesh is highlighted in Fig. 3a. In the fabricated wave guide lattice, each of these lossy sections reduced the guided intensity by (17.7 ± 0.4)%.

**Fig. 4:**
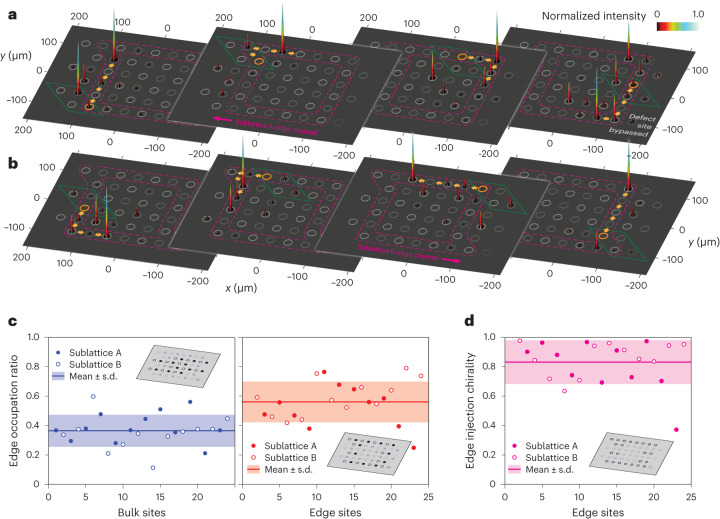
Counter-propagating topological edge states. **a**,**b**, Tracking the pair of counter-propagating topological edge channels associated with the two sublattices (dashed magenta outlines). Shown are the intensity distributions after two driving periods as observed at the output facet of the sample when injecting light into specific edge sites (highlighted in orange). Note that, while both channels flow along the outermost sites of the *x* edges (orange arrows), their paths are offset by half a unit cell (green rhombus) along the *y* edges. As a result, the clockwise-propagating channel of sublattice A shifts to bypass the deliberately induced defect in the lower right-hand corner (right panel of **a**), while the channel of sublattice B remains unaffected. **c**, EOR (fraction of the overall intensity contained in the edge channels) for a single-site edge (0.56 ± 0.03, right panel) and bulk excitations (0.36 ± 0.02, left panel). **d**, The chiral nature of topological transport in our system is clearly demonstrated by the ratio of 0.83 ± 0.03 between the intensities of the five leading versus trailing edge channel sites for each injection location evaluated for the entire set of edge excitations. The insets in **c** and **d** schematically indicate the bulk/edge excitation positions included in the analysis for the respective panels. Source data

In a first set of measurements, we investigated the light propagation in the interior of the lattice. When any individual site of an internal unit cell is excited, the injected laser beam undergoes pronounced omnidirectional spreading regardless of the targeted sublattice (Fig. 3b), as bulk transport would be suppressed only for perfect inter-sublattice transfer (Supplementary Section 4). By contrast, for our second set of measurements, single-site excitations were placed along the circumference of the array to populate the system’s helical edge states. Notably, as shown in Fig. 4a,b, the specific choice of the excited sublattice determines the orientation of the transport (clockwise for sublattice A and counterclockwise for sublattice B). We trace the path of these channels by systematically moving the point of injection along the perimeter of the lattice. In either case, the respective topological channel (indicated by dashed magenta outlines) is observed to flow along the edges and around the corners of the system. Note that both counter-propagating edge states are confined to the respective outermost rows of the lattice when propagating parallel to the *x* axis. By contrast, the ‘bearded’ edges defined by the arrangement of rhombic unit cells along the *y* axis lead to a spatial separation of the topological channels. As an illustration of this behaviour, we introduced a single-site defect in the lower right-hand corner of the fabricated wave guide array. Being located on the A sublattice, the clockwise-propagating edge state readily bypasses this defect (fourth panel in Fig. 4a) while the counterclockwise channel of sublattice B remains unaffected.

To probe the detailed characteristics of the topological transport in our system in a quantitative manner, we evaluated the edge occupation ratio (EOR) as a fraction of the total output intensity observed in the appropriate edge channels (compare with Fig. 4c). While a certain amount of light injected into bulk sites inevitably reaches the edge unit cells due to the aforementioned residual bulk diffraction (left panel, EOR_bulk_ = 0.36 ± 0.02), more than half of the light injected into the appropriate edge sites ends up in the topological channels (right panel, EOR_edge_ = 0.56 ± 0.03). While EOR represents a measure of the injection efficiency of the edge states, the helical nature of these entities has to be taken into account to evaluate the specificity of the topological transport. To this end, we calculated the chirality *χ* as the ratio between the intensities contained within the five leading versus trailing edge channel sites (Fig. 4d). With a value of *χ* = 0.83 ± 0.03, this quantity unequivocally proves that our PT-symmetric Floquet drive indeed establishes a ${{\mathbb{Z}}}_{2}$-type anomalous topological insulator whose pair of protected edges remains highly directionally selective despite the underlying complex refractive index landscape of the arrangement. Note that the non-unity coupling in steps 2 and 5 (Fig. 2a), while necessary to establish non-trivial non-Hermitian conditions, results in a certain overlap of dispersive bulk bands with the edge sites of the lattice. As such, single-site excitations inevitably yield a certain population of bulk states. Upon injection, the light contained in those bulk states propagates through the interior of the finite lattice in a non-chiral fashion, and parts of it eventually deliver some intensity to the trailing wave guides of the excited boundary state, thereby preventing experiments in finite lattices from reaching a perfect EOR_edge_ and *χ*. This bulk state excitation could be reduced by applying a broad Gaussian light injection into the boundary sites that corresponds to a narrow Gaussian excitation in k-space, which would in turn allow a more precise targeting of the corresponding boundary mode. However, such an excitation of only the clockwise or counterclockwise moving state is difficult due to the sublattice structure of the underlying unit cell allowing only both states to be excited using this injection method.

A signature of the non-Hermiticity of the system is fluctuations of the overall intensity when spectrally broad wave packets are propagating. In particular, placing sublattice-specific single-site excitations results in different total intensities. This is in stark contrast to the Hermitian regime where the orthogonality of eigenstates fundamentally precludes such a difference. In our experiments, we indeed find that the intensities resulting from excitations on sublattice A on average exceed those placed on sublattice B by approximately (19.4 ± 2.5)%, in line with the numerical value of (13.6 ± 1.2)% and substantially larger than the (1.9 ± 5.0)% observed due to the slightly non-uniform injection efficiencies in the Hermitian reference system.

In conclusion, we have presented experimental evidence of the existence of a non-Hermitian topological insulator with a real-valued energy spectrum—an important missing link between the realms of topology and non-Hermiticity. In particular, the spatiotemporal distribution of gain and loss ensures that the generalized PT symmetry is protected against spontaneous symmetry breaking; that is, the arrangement remains pseudo-Hermitian regardless of the magnitude of the applied gain–loss contrast. The lattice design allows for the two counter-propagating topological channels to be spatially separated along the ‘bearded’-type edge. We have shown that this chiral transport is robust against single-site defects, which are circumnavigated in a sublattice-specific fashion, underlining the topological nature of our system, which can be extended to all PT-symmetric topological insulators. Interestingly, our approach allows us to interpret the evolution direction as a third spatial dimension instead of as a temporal one^50^, permitting the experimental investigation of the impact of non-Hermiticities on topological singularities in higher-dimensional systems. Furthermore, while our experiments were conducted on a photonic set-up, the underlying non-Hermitian Floquet protocol can readily be generalized to any platform that allows for the implementation of discrete coupling steps and dynamic control of loss or gain, ranging from topological acoustics^51^ to topolectrical circuits^52^. Along these lines, the rich interplay between complex modulation and topology in open systems is brought into the reach of future experiments by the robust nature of the topological PT-symmetric Floquet protocol presented here, providing a pathway to exploit the dynamical properties of non-Hermitian topological systems without the instabilities entailed by complex spectra.

## Methods

### Wave guide fabrication and sample characterization

We inscribed our coupled wave guide systems with the femtosecond laser direct writing technique^48^. To this end, ultrashort laser pulses of 270 fs duration from a frequency-doubled fibre laser system (Coherent Monaco) at a wavelength of 517 nm and a repetition rate of 333 kHz were focused into a 150 mm × 25 mm × 1 mm fused silica chip (Corning 7980) by means of a microscope objective (×50, numerical aperture = 0.6). The sample was positioned with 50 nm precision by a three-axis motorized translation stage (Aerotech ALS180).

The PT-symmetric Floquet driving protocol was implemented in lattices composed of 4 × 3 unit cells in the *x*–*y* plane, for a total of 48 wave guides, and two full Floquet cycles along the propagation direction *z*. The individual single-mode wave guides are elliptical with a vertical diameter of 7 µm, a horizontal diameter of 2.5 µm and a peak refractive index contrast of 1.7 × 10^−3^ above the pristine host material’s value of 1.46. In our fully passive implementation, non-Hermiticity was implemented by introducing 100 scattering centres^23^ in each lossy section, amounting to an intensity attenuation of (17.7 ± 0.4)% as calibrated by wave guide fluorescence imaging and fine-tuned by quantitatively evaluating the input-dependent intensity output distributions of specifically designed laser-written non-Hermitian beam splitters. The scattering points were realized by exposing the desired positions to the writing laser for an additional 1.5 s, resulting in microscopic disruptions of the previously inscribed wave guide that scatter light away from it. A micrograph of the lossy coupler region is shown in Supplementary Fig. 2. The desired intra- and inter-sublattice couplings of the driving protocol were realized for wave guide separations of 11.2 µm (resulting in an intensity transfer of (98.0 ± 2)% in steps 1, 3, 4 and 6) as well as 9.8 µm and 12.4 µm (resulting in an intensity transfer of (66.8 ± 0.5)% and −(68.8 ± 0.6)% for steps 2 and 5, respectively). The lattices were characterized by injecting light at 633 nm from a continuous-wave helium–neon laser into specific lattice sites via a microscope objective (×10, numerical aperture = 0.2) and observing the resulting intensity distributions at the sample end facet with a CCD (charge-coupled device) camera (Basler ace).

The recorded output images were evaluated by extracting the relative intensities contained in each wave guide of the lattice, allowing for the calculation of the EOR (that is, the fraction of the overall intensity contained in the lattice sites belonging to the topological edge channels). Similarly, the chirality of the transport arising from single-edge-site excitations was calculated as the ratio between the five leading versus trailing edge sites relative to the point of injection.

### Reporting summary

Further information on research design is available in the Nature Portfolio Reporting Summary linked to this article.

## Online content

Any methods, additional references, Nature Portfolio reporting summaries, source data, extended data, supplementary information, acknowledgements, peer review information; details of author contributions and competing interests; and statements of data and code availability are available at 10.1038/s41563-023-01773-0.

### Supplementary information

Supplementary Information Supplementary Figs. 1–4 and Tables 1 and 2.

Reporting Summary

### Source data

Source Data Fig. 3 Source data for the intensity plots.

Source Data Fig. 4 Source data for the intensity plots, the EOR and the chirality plots.

## Data Availability

Source data are provided with this paper. All other data that support results in this Article are available from the corresponding authors upon reasonable request.

## Code availability

The codes used to generate the plots within this paper and other findings of this study can be accessed from the corresponding authors upon reasonable request.
