# Supplementary material for: Parity–time-symmetric photonic topological insulator
Source: Nat Mater. 2024 Jan 9;23(3):377–82. doi: 10.1038/s41563-023-01773-0 (PMC11349580; doi:10.1038/s41563-023-01773-0)
Supplement: Supplementary file 1 — Supplementary Figs. 1–4 and Tables 1 and 2. [file 41563_2023_1773_MOESM1_ESM.pdf]

nature materials

Article

https://doi.org/10.1038/s41563-023-01773-0

Parity–time-symmetric photonic topological insulator

In the format provided by the authors and unedited

Tabel of Contents

1. Experimental realization of the waveguide array 2
2. Fermionic time reversal symmetry of the non-Hermitian discrete Floquet model 3
3. Unitary time evolution for perfect inter-sublattice population transfer 8
4. Intensities oscillations in the non-Hermitian system 9
5. Delocalization of bulk states 10

Supplementary References 11

1. Experimental realization of the waveguide array

The waveguide array structure discussed in this paper was realized using the femtosecond laser writing technique described in (*48*). The focused laser pulses induce a permanent refractive index increase in the host material fused silica, allowing for the direct inscription of extended pathways for light. The electric field envelope of the light is described by the paraxial Helmholtz equation

$$i\bar{\lambda}\frac{\partial\, E(x,y,z)}{\partial z} = -\left(\frac{\bar{\lambda}^2}{2n_0}\left[\frac{\partial^2}{\partial x^2} + \frac{\partial^2}{\partial y^2}\right] + \Delta n(x,y,z)\right) E(x,y,z) \qquad \text{(S.1)}$$

where $\bar{\lambda} = \lambda/2\pi$ denotes the reduced wavelength and Δn describes the reflective index landscape compared to the bulk refractive index n_0. This equation is mathematically equivalent to the Schrödinger equation governing electron dynamics with the exception that in equ. (S.1) the propagation direction of the light z plays the role of time. Hence, by changing the refractive index profile, photonic waveguides can be used to model electronic systems of different potentials. Using this analogy, one can also realize different lattices by arranging waveguides periodically in the x, y-plane where the light evolution in the propagation direction is governed by the tight binding equation (*1*). To observe the propagation of an initial excitation through a waveguide lattice one can simply measure the intensity distribution of the light at the end of the fused silica chip called end facet.

The loss regions were realized following (*23*) using microscopic scattering points (Fig. S1). To write these scattering points, the writing laser stopped at specific points thereby producing small disruptions in the waveguide. These microscopic dots scatter some of the light in the waveguide away from it such that it is effectively lost to the system. To produce scattering points with similar properties the waveguide was written first and the scattering points were added to it afterwards.

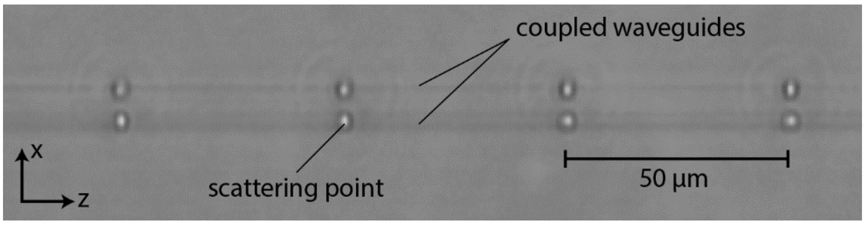

Figure S1: Microscope image of a part of a lossy coupling region of the described protocol containing the scattering points. The distance between two scattering points is approximately 50 µm.

2. Fermionic time reversal symmetry of the non-Hermitian discrete Floquet model

Periodically modulated systems satisfying the Schrödinger equation

$$i\frac{d\Psi(z)}{dz} = H(z)\Psi(z) \tag{S.2}$$

with $H(z+L) = H(z)$ can be treated using Floquet's theorem which leads to solutions that consist of a periodic part multiplied by a phase $\Psi(z) = e^{-i\varepsilon z}\Phi(z)$ with $\Phi(z+L) = \Phi(z)$. The quasi-energies ε are periodic with $2\pi/L$. Conveniently, the quasi-energy band structure can be computed from the time evolution operator $U(L) = \mathcal{P}\exp\left\{-i\int_0^L H(z)dz\right\}$ that connects a state at distance L with an initial state, $\Psi(L) = U(L)\Psi(0)$. $\mathcal{P}$ denotes the path-ordering operator. The eigenvalues of this operator are then $e^{-i\varepsilon L}$. To calculate the quasi-energies from these eigenvalues, the branch of the complex logarithm is chosen such that $\varepsilon \in \left(-\frac{\pi}{L}, \frac{\pi}{L}\right]$.

The model discussed in our paper consists of six distinct time steps with piecewise constant couplings $c(z)$ and on-site potentials $g(z)$. The Hermitian version of this model is based on the anomalous $\mathbb{Z}_2$-driving protocol described in (*40*). We introduce non-Hermiticity to the system by dynamically distributing on-site gain and loss throughout the driving cycle (See Fig. S2 for an extended schematic of the three-dimensional waveguide array).

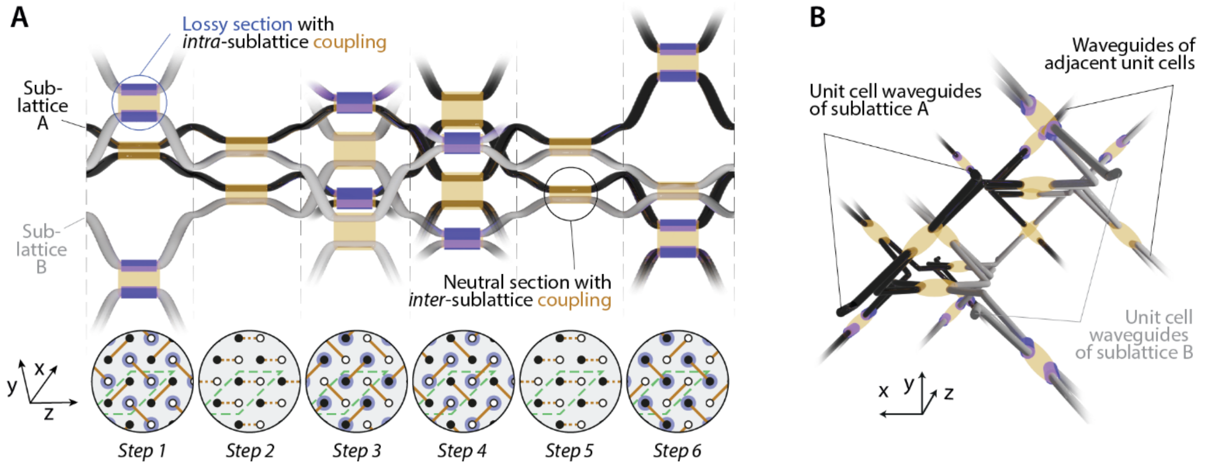

Figure S2: Waveguide-based passive-PT implementation of the non-Hermitian anomalous driving protocol. (A) Extended version of Fig. 3 of the main text showing the unit cell waveguides as well as sections of waveguides of adjacent unit cells they couple to. (B) Front view of the implemented waveguide array.

The single-step Hamiltonians in momentum space are shown in Tab. S1 with $k_1 = a(k_x + k_y)/2$ and $k_2 = a(k_x - k_y)/2$. By sequentially applying the respective single-step time evolution operators (Tab. S2), one can express the time evolution operator as $U(\gamma, L) = U_6 U_5 U_4 U_3 U_2 U_1$. For all couplings, the time evolution operator can be written as $U(\gamma, L) = M^{-1}(\gamma) U(\gamma = 0, L) M(\gamma)$ with $M(\gamma) = \mathrm{diag}(\mathrm{e}^{2\gamma}, 1, \mathrm{e}^{2\gamma}, 1)$. Due to this relation, the eigenvalues of $U(\gamma, L)$ remain strictly real regardless of the amount of gain/loss as can be seen for instance for the analytically calculated quasi-energy band structure for the case $c = c'$ and $L = 6$

$$\varepsilon = \pm \arccos\left\{\frac{1}{32}\left[22 + cos(2c) + 10\, cos(4c) - cos(6c) + 32\, cos^2(c)\, cos(4k_y)\, sin^4(c) - 16\left(cos(2k_x) + 2\, cos^2(k_x)\, cos(2k_y)\right) sin^2(2c)\right]\right\} \tag{S.3}$$

which is independent of γ.

Table S1: Single-step Hamiltonians of the non-Hermitian driving protocol in momentum space ($k_1 = a(k_x + k_y)/2$ and $k_2 = a(k_x - k_y)/2$). with gain/loss γ and intra sublattice coupling constants c and inter-sublattice couplings c' .

**Driving Protocol**	**Single-Step Hamiltonian**
Step 1: $\boldsymbol{0 \le z \le \frac{L}{6}}$	$H_1 = \begin{pmatrix} i\gamma & 0 & ce^{ik_1} & 0 \\ 0 & -i\gamma & 0 & ce^{ik_2} \\ ce^{-ik_1} & 0 & i\gamma & 0 \\ 0 & ce^{-ik_2} & 0 & -i\gamma \end{pmatrix}$
Step 2: $\boldsymbol{\frac{L}{6} < z \le \frac{2L}{6}}$	$H_2 = \begin{pmatrix} 0 & c'e^{ik_x} & 0 & 0 \\ c'e^{-ik_x} & 0 & 0 & 0 \\ 0 & 0 & 0 & c'e^{ik_x} \\ 0 & 0 & c'e^{-ik_x} & 0 \end{pmatrix}$
Step 3: $\boldsymbol{\frac{2L}{6} < z \le \frac{3L}{6}}$	$H_3 = \begin{pmatrix} -i\gamma & 0 & ce^{-ik_1} & 0 \\ 0 & i\gamma & 0 & ce^{-ik_2} \\ ce^{ik_1} & 0 & -i\gamma & 0 \\ 0 & ce^{ik_2} & 0 & i\gamma \end{pmatrix}$
Step 4: $\boldsymbol{\frac{3L}{6} < z \le \frac{4L}{6}}$	$H_4 = \begin{pmatrix} i\gamma & 0 & ce^{-ik_2} & 0 \\ i & -i\gamma & 0 & ce^{-ik_1} \\ ce^{ik_2} & 0 & i\gamma & 0 \\ 0 & ce^{ik_1} & 0 & -i\gamma \end{pmatrix}$
Step 5: $\boldsymbol{\frac{4L}{6} < z \le \frac{5L}{6}}$	$H_5 = \begin{pmatrix} 0 & -c'e^{ik_x} & 0 & 0 \\ -c'e^{-ik_x} & 0 & 0 & 0 \\ 0 & 0 & 0 & -c'e^{ik_x} \\ 0 & 0 & -c'e^{-ik_x} & 0 \end{pmatrix}$
Step 6: $\boldsymbol{\frac{5L}{6} < z \le L}$	$H_6 = \begin{pmatrix} -i\gamma & 0 & ce^{ik_2} & 0 \\ 0 & i\gamma & 0 & ce^{ik_1} \\ ce^{-ik_2} & 0 & -i\gamma & 0 \\ 0 & ce^{-ik_1} & 0 & i\gamma \end{pmatrix}$

Table S2: Single-step time evolution operators of the non-Hermitian protocol for the single-step Hamiltonians of Tab. S1 in momentum space for $L = 6$.

**Single-Step Time Evolution Operators**
$U_1 = \begin{pmatrix} e^{\gamma}\cos(c) & 0 & -i\sin(c)\, e^{ik_1+\gamma} & 0 \\ 0 & e^{-\gamma}\cos(c) & 0 & -i\sin(c)\, e^{ik_2-\gamma} \\ -i\sin(c) e^{-ik_1+\gamma} & 0 & e^{\gamma}\cos(c) & 0 \\ 0 & -i\sin(c)\, e^{-ik_2-\gamma} & 0 & e^{-\gamma}\cos(c) \end{pmatrix}$
$U_2 = \begin{pmatrix} \cos(c') & -i\sin(c')\, e^{ik_x} & 0 & 0 \\ -i\sin(c')\, e^{-ik_x} & \cos(c') & 0 & 0 \\ 0 & 0 & \cos(c') & -i\sin(c')\, e^{ik_x} \\ 0 & 0 & -i\sin(c')\, e^{-ik_x} & \cos(c') \end{pmatrix}$
$U_3 = \begin{pmatrix} e^{-\gamma}\cos(c) & 0 & -i\sin(c)\, e^{-ik_1-\gamma} & 0 \\ 0 & e^{\gamma}\cos(c) & 0 & -i\sin(c)\, e^{-ik_2+\gamma} \\ -i\sin(c)\, e^{ik_1-\gamma} & 0 & e^{-\gamma}\cos(c) & 0 \\ 0 & -i\sin(c)\, e^{ik_2+\gamma} & 0 & e^{\gamma}\cos(c) \end{pmatrix}$
$U_4 = \begin{pmatrix} e^{\gamma}\cos(c) & 0 & -i\sin(c)\, e^{-ik_2+\gamma} & 0 \\ 0 & e^{-\gamma}\cos(c) & 0 & -i\sin(c)\, e^{-ik_1-\gamma} \\ -i\sin(c)\, e^{ik_2+\gamma} & 0 & e^{\gamma}\cos(c) & 0 \\ 0 & -i\sin(c)\, e^{ik_1-\gamma} & 0 & e^{-\gamma}\cos(c) \end{pmatrix}$
$U_5 = \begin{pmatrix} \cos(c') & i\sin(c')\, e^{ik_x} & 0 & 0 \\ i\sin(c')\, e^{-ik_x} & \cos(c') & 0 & 0 \\ 0 & 0 & \cos(c') & i\sin(c')\, e^{ik_x} \\ 0 & 0 & i\sin(c')\, e^{-ik_x} & \cos(c') \end{pmatrix}$
$U_6 = \begin{pmatrix} e^{-\gamma}\cos(c) & 0 & -i\sin(c)\, e^{ik_2-\gamma} & 0 \\ 0 & e^{\gamma}\cos(c) & 0 & -i\sin(c)\, e^{ik_1+\gamma} \\ -i\sin(c)\, e^{-ik_2-\gamma} & 0 & e^{-\gamma}\cos(c) & 0 \\ 0 & -i\sin(c)\, e^{-ik_1+\gamma} & 0 & e^{\gamma}\cos(c) \end{pmatrix}$

The Hermitian model with $\gamma = 0$ satisfies fermionic time reversal symmetry which, due to the periodicity of the protocol, can be written as

$$H_{7-n}(-\boldsymbol{k}) = \mathcal{T} H_n^*(\boldsymbol{k}) \mathcal{T}^{-1} \tag{S.4}$$

where $*$ denotes complex conjugation and the time-reversal operator

$$\mathcal{T} = \begin{pmatrix} 0 & -i & 0 & 0 \\ i & 0 & 0 & 0 \\ 0 & 0 & 0 & -i \\ 0 & 0 & i & 0 \end{pmatrix} \quad .$$

In combination with complex conjugation, this operator therefore connects steps n and $7-n$, and exchanges the two sublattices in the unit cell such that in the time-reversal symmetric case, on-site terms satisfy $g_n^{\bullet} = (g_{7-n}^{\circ})^*$ and vice-versa. The directional hoppings transform accordingly such that inter-sublattice couplings are invariant, while intra-sublattice couplings keep their orientation but connect opposite sublattices. This symmetry operator, as discussed in *(53),* allows the realization of fermionic and bosonic TRS while avoiding long-range hoppings which are not experimentally feasible. The presence of non-Hermiticity ($\gamma \neq 0$) allows for a second type of time-reversal symmetry (*25,54*),

$$H_{7-n}(-\boldsymbol{k}) = \mathcal{T} H_n^T(\boldsymbol{k}) \mathcal{T}^{-1} \,, \tag{S.5}$$

to be defined based on transposition (T). This symmetry maps couplings in the same way as the first type of TRS, but results in a different relation for the on-site terms $g_n^{\bullet} = g_{7-n}^{\circ}$ and vice-versa. These two definitions are fully equivalent in Hermitian systems (which fulfill $H^* = H^T$). Whereas Eq. (S.4) no longer holds in our non-Hermitian model, Eq. (S.5) remains fulfilled and serves to protect a pair of counter-propagating boundary states.

In addition to the TRS, we can find an antilinear self-inverse symmetry operator that can act as a generalized PT-symmetry operator. To obtain a simple representation of the symmetry operator we first transform the single step Hamiltonians using the transformation $\Phi = \mathrm{diag}\left(e^{i\varphi}, 1, e^{i\varphi}, 1\right)$ with $\varphi = \frac{\pi}{2} - k_x$ and perform the transformation $\Theta = [\frac{1}{\sqrt{2}} \mathbb{I} \otimes (\mathbb{I} + i\sigma_x)]$ with the Pauli matrix σ_x on the effective Hamiltonian. The effective Hamiltonian H'_{eff} than takes the form

$$H'_{\mathrm{eff}} = \begin{pmatrix} i\kappa(k_x, k_y, c, \gamma) & q(k_x, k_y, c, \gamma) & h(k_x, k_y, c) & 0 \\ q^*(k_x, k_y, c, \gamma) & -i\kappa(k_x, k_y, c, \gamma) & 0 & h(k_x, k_y, c) \\ h^*(k_x, k_y, c) & 0 & -i\kappa(k_x, k_y, c, \gamma) & -q(k_x, k_y, c, \gamma) \\ 0 & h^*(k_x, k_y, \text{[illegible]}) & -q^*(k_x, k_y, c, \gamma) & i\kappa(k_x, k_y, c, \gamma) \end{pmatrix}$$

with eigenvalues $\varepsilon(k_x, k_y, c) = \pm\sqrt{|h|^2 + |q|^2 - \kappa^2}$ and eigenvectors $\Psi_{1,3}(k_x, k_y, c, \gamma) = \left(\{\varepsilon + i\kappa\} \cdot \frac{1}{h^*}, \frac{q^*}{h^*}, 1, 0\right)^T$ and $\Psi_{2,4}(k_x, k_y, c, \gamma) = \left(\frac{q}{h^*}, \{\varepsilon - i\kappa\} \cdot \frac{1}{h^*}, 0, 1\right)^T$ where lower-case Greek letters denote real values and Latin letters denote complex values. Here, eigenvectors Ψ_1 and Ψ_2 as well as Ψ_3 and Ψ_4 are eigenvectors to the same degenerate real eigenvalue. This effective Hamiltonian is invariant under the symmetry operation $(SK)H'_{eff}(SK) = H'_{eff}$ with K denoting complex conjugation and $S = \begin{pmatrix} 0 & \frac{h}{h^*} & 0 & 0 \\ \frac{h}{h^*} & 0 & 0 & 0 \\ 0 & 0 & 0 & 1 \\ 0 & 0 & 1 & 0 \end{pmatrix}$ being a unitary operator. The operator A=SK is antilinear and self-inverse with eigenvalues ± 1 and eigenvectors $\Phi_{1,2} = \Psi_1 \pm \Psi_2$ and $\Phi_{3,4} = \Psi_3 \pm \Psi_4$ which are also eigenvectors of the effective Hamiltonian due to the degeneracy of the eigenvalues. Therefore, the operator SK can be regarded as a generalized PT-symmetry operator which shares the same set of eigenvalues and commutes with the effective Hamiltonian.

3. Unitary time evolution for perfect inter-sublattice population transfer

In our system, we considered equal intra-sublattice couplings for steps 1,3,4 and 6 and chose partial coupling for inter-sublattice hoppings c'. Perfect population transfer ($c = \pm 3\pi/L$) would result in a trivial and unitary time evolution operator $U(L) = \pm\mathbb{I}$ and a correspondingly flat quasi-energy band structure in line with Eq. (S.3). If, on the other hand, the inter-sublattice couplings are set to unity and the intra-sublattice couplings remain partial, the system would still exhibit unitary time evolution. In this case, the single-step Floquet operators (cf. Tab. S2) for steps 2 and 5 become

$$U_{2,5}^{\text{perf}} = \mp i\frac{L}{6}\begin{pmatrix} 0 & e^{ik_x} & 0 & 0 \\ e^{-ik_x} & 0 & 0 & 0 \\ 0 & 0 & 0 & e^{ik_x} \\ 0 & 0 & e^{-ik_x} & 0 \end{pmatrix}. \tag{S.6}$$

The Floquet operators of steps 1,3,4 and 6 can be separated into a matrix $N(\pm\gamma) = \text{diag}(e^{\pm i\gamma}, e^{\mp i\gamma}, e^{\pm i\gamma}, e^{\mp i\gamma})$ that contains the non-Hermiticities and a matrix U_n^{Hop} describing the hopping of the step (see Tab. S2). For these steps, N and U_n^{Hop} commute, while N and $U_{2,5}$ do not. Instead, in the case of perfect population transfer, they satisfy the relation $N(\gamma)U_{2,5}^{\text{perf}} = U_{2,5}^{\text{perf}}N(-\gamma)$. Therefore, the non-Hermiticities would cancel out in $U(L)$, and the time evolution would be unitary despite the existence of non-Hermiticities in the system.

In the experiment we chose an entirely passive implementation of the driving protocol by adding a global uniform loss term to the system corresponding to $+\gamma \rightarrow 0$ and $-\gamma \rightarrow -2\gamma$. Additionally, the neutral inter-sublattice coupling steps 2 and 5 are realized without additional losses in order to boost the overall transmission of the experimental system. The constant shift along the negative imaginary axis of the spectrum faithfully preserves the dynamics of the system such that the key features of the PT-symmetric model could be experimentally observed.

4. Intensities oscillations in the non-Hermitian system

An arbitrary state can always be written as the superposition of the linearly independent eigenstates Φ_i of a system

$$\Psi = \sum_i a_i\, e^{-i\varepsilon_i z}\Phi_i \qquad \text{(S.7)}$$

where a_i are complex coefficients. If the system is Hermitian and the time evolution therefore unitary, the eigenstates are orthogonal $\Phi_i\Phi_j^* = \delta_{ij}$. In contrast, for a non-Hermitian system with a non-unitary time evolution, the eigenstates become non-orthogonal (*55*). As a consequence, the intensity I of arbitrary states is no longer constant, but rather oscillates as a function of time:

$$I = \Psi\Psi^* = \sum_{i,j} a_i a_j^*\, e^{-i(\varepsilon_i-\varepsilon_j)z}\Phi_i\Phi_j^*$$

$$= \sum_i a_i a_i^*\, \Phi_i\Phi_i^* + \sum_{i\neq j} a_i a_j^* e^{-i(\varepsilon_i-\varepsilon_j)z}\Phi_i\Phi_j^* \qquad \text{(S.8)}$$

In Hermitian systems, the second term vanishes such that the intensity of arbitrary states is constant, while it oscillates in non-Hermitian ones with terms proportional to $e^{-i(\varepsilon_i-\varepsilon_j)z}$.

We verified this behavior in our system by conducting long-range propagation simulations of single-site bulk excitation in or system. As shown in Fig. S3 for the example of sublattice A, despite an apparently random fluctuation between subsequent driving cycles, no long-term decay or increase is visible even after 10^5 Floquet cycles. However, the intensity of eigenstates Φ_i does not fluctuate and stays constant over time.

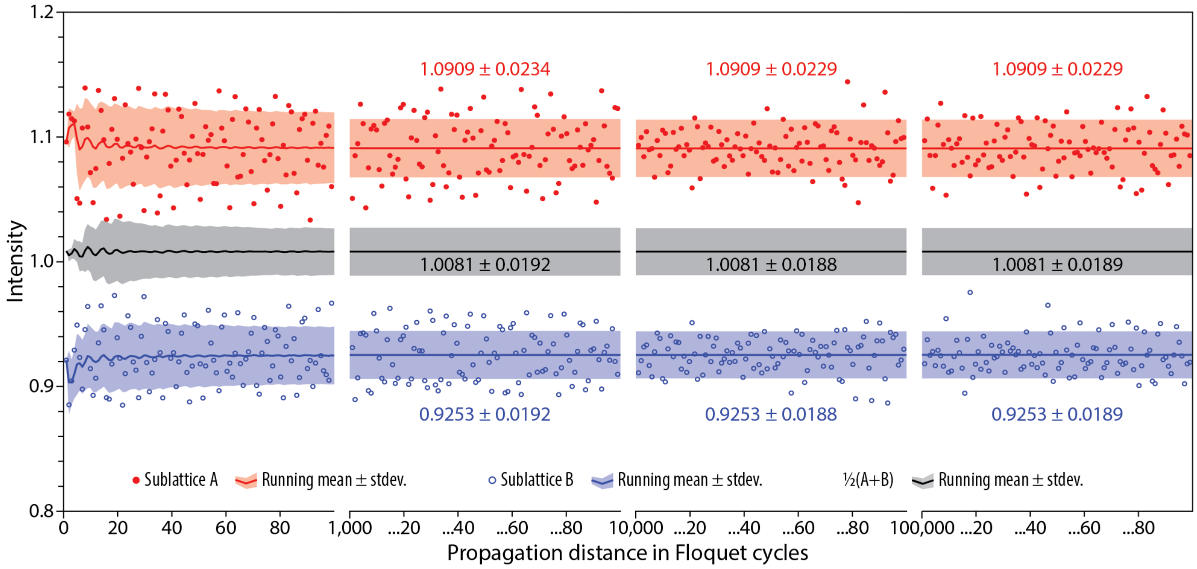

Figure S3: Long-term behavior of intensity fluctuations. Simulated intensity of a bulk excitation on sublattice A as a function of the number Floquet cycles for the PT-symmetric system with a gain/loss coefficient of $|\gamma| = 10\%$. While the intensity in both sublattices fluctuates randomly from cycle to cycle, its running mean and standard deviation soon converge towards long-term stable values.

5. Delocalization of bulk states

In this section we further want to study the bulk localization behavior as a function of the coupling strength in steps 2 and 5. Figure S4a shows the calculated inverse participation ratio (IPR) of the four bulk excitations in Fig. 3 of the main manuscript for perfect intra-sublattice coupling and varying degrees of the inter-sublattice couplings in the case of $\gamma = 0$ (Hermitian case) for the non-Hermitian passive system realized in the experiment after two driving cycles. For a perfect transfer of population between sublattices, the bulk states become fully localized ($IPR = 1$). When the inter-sublattice coupling is reduced, the IPR sharply drops, indicating a rapid delocalization of bulk states even though the system remains topological.

For the non-Hermitian configuration (Fig. S4b), this behavior is somewhat distorted and the IPR becomes strongly dependent on the initially excited waveguide and its sublattice. As 100% inter-sublattice coupling renders non-Hermitian the system trivially unitary, the experiments were conducted at an intermediate value of approximately 67%, allowing us to observe the protected edge states of the system despite an absence of bulk localization.

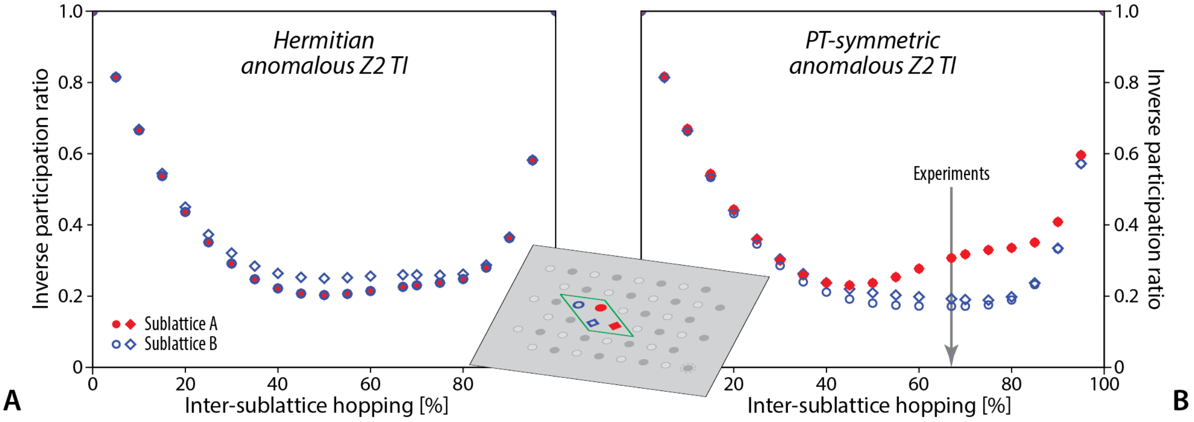

Figure S4: Bulk localization vs inter-sublattice hopping. Shown is the calculated inverse participation ratio (IPR) after two driving periods for the four different locations of bulk excitations shown in Fig. 3 for the **(A)** Hermitian case and **(B)** non-Hermitian case (right) for different inter-sublattice coupling strengths. An IPR of 1 represents perfect suppression of bulk transport. The insert schematically indicates the position of the unit cell and its four excitation positions included in the analysis.

Supplementary references

53. Höckendorf, B., Alvermann, A. & Fehske, H. Universal driving protocol for symmetry-protected Floquet topological phases, *Phys. Rev. B* **99**, 245102 (2019)
54. Zhou, H. & Lee, J. Y. Periodic table for topological bands with non-Hermitian symmetries, *Phys. Rev. B* **99**, 235112 (2019)
55. Moiseyev, N. *Non-Hermitian Quantum Mechanics.* Cambridge University Press, Cambridge (2011)
